# Validity and reliability of medication adherence scale in FMF

**DOI:** 10.1186/1546-0096-13-S1-P112

**Published:** 2015-09-28

**Authors:** BE Fidanci, S Yesilkaya, C Acikel, A Ozden, D Simsek, F Yildiz, B Kisacik, M Sayarlioglu, S Akar, S Senel, M Tunca, S Yavuz, A Tufan, A Berdeli, AM Onat, A Gul, B Goker, T Kasifoglu, H Direskeneli, S Erten, G Ozcelik, F Gok, S Ozen, E Demirkaya

**Affiliations:** 1 FMF Arthritis Vasculitis and Orphan Disease Research in Paediatric Rheumatology (FAVOR), Ankara, Turkey

## Objective

The optimal level of adherence necessary to achieve acceptable disease and quality-of-life outcomes for patients is not known. In order to identify these optimal levels, we need reliable and valid measures of adherence. Medication Adherence Scale in FMF (MASIF) is an instrument designed to measure adherence to treatment in children with Familial Mediterranean Fever (FMF). We have developed this scale for children with FMF and found valid and reliable. In this study, it was aimed to assess the validity and reliability of this adherence scale for medical treatment in adult FMF patients.

## Methods

This study is multicentre and 14 centers participated to the study. Patients with FMF using medication at least for 6 months and accepted to participate constituted the sample of the study. Besides “Medication Adherence Scale in FMF Patients (MASIF)”, “Data collection forms about the sociodemographic and medical information (demographic, clinical and laboratory findings) of patients”, and “Morisky Medication Adherence Scale (MMAS)” were used as data collection instruments. Morisky medication adherence scale was used as a gold standard in order to evaluate the criterion validity of MASIF. We assessed the validity of the adult version of the MASIF using the Outcome Measures in Rheumatoid Arthritis Clinical Trials (OMERACT) filter.

## Results

There were 133 patients with FMF enrolled for the validation of the study. The median age of the patients (n=133) was 28.60 years (min.18.12-max.71.34) and 52.6% of them were female. The median number of the attack frequency was 13.50 (min. 0-max 99) in a year and 57.9% of the participants had irregular attacks. For internal consistency, Cronbach's alpha was 0,764 for MASIF adult version. Also, there was a positive and significant correlation between test and retest score (t=0.971; p=0.340). For the “criterion validity” the correlation with Morisky and MASIF was evaluated (r=0.530, p=0.000) and for the “structure” validity, factor analyzes and Kaiser-Meyer-Olkin tests were performed. After these tests, MASIF was found as a valid and reliable instrument. MASIF consists of 18 items and 4 sub-dimensions: knowledge about the medication, adherence to the treatment, barriers to drug use, factors that may increase compliance. The participants answered each item on a Likert scale (1=strongly agree, 2=agree, 3=no idea, 4=disagree, 5=strongly disagree). The total score ranged from 18 to 90. A high score showed a good adherence to treatment. The cut-off point was determined as 55 points. A point over 55 was accepted as “good medication adherence” and a point less than 55 was considered as “bad medication adherence"

## Conclusion

Approximately 10-15% of patients with FMF are non-responders but it was claimed that in fact they are non-compliers that causes these patients receive unnecessary biologic agent treatment procedures, which are expensive and have some serious adverse effects. This scale will provide assessment and follow up of adherence to treatment patients and determine whether the patient is non-responders or non-compliers. It may help to determine the non-compliance and prevent unnecessary and expensive biologic agents.

